# Temporal dynamics of ovine airway epithelial cell differentiation at an air-liquid interface

**DOI:** 10.1371/journal.pone.0181583

**Published:** 2017-07-26

**Authors:** Nicky O’Boyle, Erin Sutherland, Catherine C. Berry, Robert L. Davies

**Affiliations:** 1 Institute of Infection, Immunity and Inflammation, College of Medical, Veterinary and Life Sciences, University of Glasgow, Glasgow, United Kingdom; 2 Institute of Molecular Cell and Systems Biology, College of Medical, Veterinary and Life Sciences, University of Glasgow, Glasgow, United Kingdom; University of Alabama at Birmingham, UNITED STATES

## Abstract

The respiratory tract and lungs are subject to diverse pathologies with wide-ranging implications for both human and animal welfare. The development and detailed characterization of cell culture models for studying such forms of disease is of critical importance. In recent years the use of air-liquid interface (ALI)-cultured airway epithelial cells has increased markedly, as this method of culture results in the formation of a highly representative, organotypic *in vitro* model system. In this study we have expanded on previous knowledge of differentiated ovine tracheal epithelial cells by analysing the progression of differentiation over an extensive time course at an ALI. We observed a pseudo-stratified epithelium with ciliation and a concurrent increase in cell layer thickness from 9 days post-ALI with ciliation approaching a maximum level at day 24. A similar pattern was observed with respect to mucus production with intensely stained PAS-positive cells appearing at day 12. Ultrastructural analysis by SEM confirmed the presence of both ciliated cells and mucus globules on the epithelial surface within this time-frame. Trans-epithelial electrical resistance (TEER) peaked at 1049 Ω × cm^2^ as the cell layer became confluent, followed by a subsequent reduction as differentiation proceeded and stabilization at ~200 Ω × cm^2^. Importantly, little deterioration or de-differentiation was observed over the 45 day time-course indicating that the model is suitable for long-term experiments.

## Introduction

The primary role of the respiratory system is to conduct air through the nasopharynx, via the trachea, bronchi and bronchioles, into the alveoli for gaseous exchange. During the process of inhalation the respiratory system is exposed to a variety of particulates including bacteria, viruses, and pollutants [1–3]. The airway epithelium lines the luminal surface of the nasopharynx, trachea, bronchi and bronchioles. It represents the primary point of interaction between inhaled foreign organisms and the host and as such the epithelium has evolved diverse defense mechanisms in order to maintain a virtually sterile environment in the small conducting airways [2, 3]. Successful clearance of particulates depends on an intact, fully functioning epithelial barrier with a complex cellular organization, whereby diverse cell types co-operate in order to maintain airway homeostasis [4]. Airway epithelia consist of a self-regenerating, pseudo-stratified layer with basal progenitor cells, mucus producing goblet cells, actively beating ciliated cells, sensory brush cells and secretory club cells (also known as Clara cells) [5–9]. In the large airways, a principle activity of the epithelium is to carry out mucociliary clearance by entrapping organisms and other inhaled particles in goblet cell-derived mucus before propelling the mucus anteriorly towards the esophagus via the co-ordinated beating action of ciliated cells [10].

When studying the interaction between microorganisms and host tissues it is important that the tissue complexity is considered. A number of primary and immortalized cell lines have been used in conventional submerged monolayer culture for the study of respiratory pathogens [11–17]. However, submerged monolayer cultures poorly reflect the complex architecture of the airway epithelium and in many cases fail to develop the critical mucociliary differentiation phenotype of the *in vivo* respiratory tract [18, 19]. An early study identified that by using a mechanically supported cell culture substrate, epithelial cells could be cultured at an air-liquid interface (ALI), more akin to the *in vivo* environment, yielding vastly improved levels of differentiation [20]. It was later found that finely tuned levels of epidermal growth factor and retinoic acid could further improve the level of differentiation observed [19]. Since then, there has been a marked increase in the number of studies using differentiated ALI cultures, due to the fact that differentiated ALI cultures form an organotypic cell layer containing all of the major cell types, which closely matches the morphological phenotype and expression profile of the native epithelium [19, 21–23]. Airway ALI cultures have been employed for diverse applications in fields including toxicology, pharmacokinetics, pathology, virology and bacteriology [24–28].

While a great deal of effort has been expended in developing and characterizing the culture of human airway epithelial cells, detailed knowledge of other mammalian airway culture systems is somewhat lacking. Respiratory disease is one of the principle causes of economic loss in the livestock industry [29]. Detailed insight into the pathogenic mechanisms of the organisms responsible for such infections has been hampered by the lack of appropriate infection models. In 2015, sheep production provided the third largest contribution to the UK meat industry, behind cattle and poultry, with £1.1 billion being generated [30]. A number of important sheep respiratory pathogens have been identified including *Pasteurella multocida*, *Mannheimia haemolytica*, *Bibersteinia trehalosi*, *Histophilus somni*, *Mycoplasma ovipneumoniae* and respiratory syncytial virus [31–36]. Many of these organisms cause infection outbreaks and are readily spread not only between sheep but also to other livestock such as cattle and goats [37, 38]. Developing an understanding of how these organisms colonize the airway epithelium will form a crucial part of our understanding of disease progression and transmission dynamics. To this end, we sought to develop a sheep-derived airway epithelial ALI culture model.

Sheep airway epithelia have been successfully differentiated at ALI by a number of groups in recent years [39–43]. Two of these studies described the use of ALI cultures as infection models to study the sheep pathogen *M*. *ovipneumoniae* [40, 42]. However, detailed characterization of the model has not yet been achieved both in a temporal context (differentiation over time) and a spatial context (cellular organization within the tissue). The differentiation of ALI cultures is a complex and dynamic process involving a step-wise progression consisting of cellular attachment, followed by squamous proliferation, cell layer thickening and polarization, mucociliary differentiation and finally in many cases de-differentiation characterized by reductions in ciliation, mucus production and cell death [19, 21, 44]. A number of studies have detailed important temporal aspects of both differentiation and indeed de-differentiation/deterioration of human ALI cultures [21, 22, 45]. One of these studies highlighted the requirement for detailed characterization and determination of an optimum window for use of the ALI culture [21]. As such we aimed to enhance current knowledge of ovine airway epithelial ALI culture by conducting a detailed time-course over 42 days of growth and analyzing a variety of important markers of differentiation. This served to identify a window within which the cell layer was optimally differentiated, thereby improving the utility and applicability of the model for future infection studies.

## Materials and methods

### Ovine tracheal cell isolation, expansion and culture at ALI

Tracheae from freshly slaughtered sheep were obtained from a local abattoir (Sandyford Abattoir Co, Paisley, United Kingdom) and transported to the laboratory in chilled PBS containing 1% (v/v) penicillin-streptomycin and 1% (v/v) Fungizone. All subsequent media were also supplemented with penicilillin-streptomycin and Fungizone. Samples of native tracheal tissue were fixed in 2% (w/v) formaldehyde overnight to allow for histological comparison of ALI cultures with *ex vivo* tissue. Epithelial tissue was dissected from the underlying cartilage and digested overnight at 4°C in Dulbecco’s modified minimal Eagle’s medium (DMEM)/Ham’s F12 (1:1) containing 10 μg ml^-1^ DNase, 1 mg ml^-1^ dithiothreitol and 1 mg ml^-1^ protease XIV from *Streptomyces griseus* (Sigma-Aldrich). Digestion was halted by the addition of 10% (v/v) fetal calf serum (FCS). Tissue pieces were rinsed thoroughly to remove loosely attached cells and to homogenize the cell suspension. The cells were strained through a 70 μm cell strainer, collected by centrifugation and washed with DMEM/Ham’s F12 (1:1) with 10% (v/v) FCS. The cells were again centrifuged and resuspended in airway epithelial growth medium (AEGM) (Promocell). Viability of the extracted cells was assessed by Trypan Blue exclusion and was typically found to be approximately 90%. Tissue culture flasks (75 cm^2^) were seeded with 1.0 × 10^7^ cells per flask and cultures were expanded to approximately 70% confluency (~7 days). Epithelial cells were routinely cultured in a Heraeus 150i incubator at 37°C, 5% CO_2_, 14% O_2_. At this point the cells were trypsinized and seeded onto high pore density, translucent Thincerts (Greiner #665640, pore diameter 0.4 μm, 1 x 10^8^ pores cm^-2^) at a density of 2.5 × 10^5^ cells per insert in 0.5 ml AEGM. For bright field microscopy and movie capture of beating cilia low pore density, transparent Thincerts were employed (Greiner #665641, pore diameter 0.4 μm, 2 x 10^6^ pores cm^-2^). One milliliter volumes of AEGM were added to the basal compartment. Trans-epithelial electrical resistance (TEER) was monitored on a daily basis using an EVOM2 epithelial voltmeter with STX2 electrode (World Precision Instruments) and cells were washed and fed every two to three days. Once the TEER reached 200 Ω × cm^2^ in submerged culture, the ALI was established by removing all apical medium, thereby exposing the luminal surface to the atmosphere (day 0 post-ALI). Following the formation of the ALI the cells were fed exclusively from the basal compartment with complete ALI medium consisting of DMEM/AEGM base medium [1:1] supplemented with the following growth factors: 100 nM retinoic acid, 10 ng ml^-1^ epidermal growth factor, 5 μg ml^-1^ insulin, 500 ng ml^-1^ hydrocortisone, 500 ng ml^-1^ epinephrine, 6.7 ng ml^-1^ triiodo-thyronine and 10 μg ml^-1^ transferrin. A 50:50 mix of complete ALI medium and AEGM was employed for apical and basal feeding approximately half way through the submerged growth phase to allow for a gradual transition to ALI conditions. Apical surface washing, basal feeding and TEER measurements were carried out every 2 to 3 days until day 42 post-ALI.

### Immunofluorescence microscopy

Samples were taken at 3 day intervals starting from 3 days prior to establishment of the ALI (i.e., day -3). At each time-point cultures were fixed by adding 4% (w/v) paraformaldehyde to the apical surface and incubating at room temperature for 15 min. Samples were washed with 1 ml PBS and stored in PBS until completion of the time course. Samples were then incubated in 0.5 ml permeabilization buffer (PBS with 0.5% [v/v] Triton X-100, 100 mg ml^-1^ sucrose, 4.8 mg ml^-1^ HEPES, 2.9 mg ml^-1^ NaCl and 600 μg ml^-1^ MgCl_2_, pH 7.2) for 10 min. The apical surfaces were washed three times for 3 min with PBS and blocked with PBST (PBS with 0.1% Tween-20) containing 10% (v/v) normal goat serum and 1% (w/v) BSA for 1 h. The ALI cultures were incubated for 1 h with primary antibodies diluted in blocking buffer to the following concentrations: rabbit anti-β-tubulin antibody (Abcam #ab6046) 1:200, mouse anti-ZO1 antibody (Thermofisher #33–9100) 1:50 and Jacalin-FITC lectin (Vector #FL-1151) 1:25. ALI cultures were washed (three times for 3 min) with PBST to remove unbound antibody and incubated in goat anti-rabbit-Alexa488 (Thermofisher #A-11034) or goat anti-mouse-Alexa488 (Thermofisher #A-11001) at a 1:400 dilution in blocking buffer for 1 h. The ALI cultures were washed three times for 3 min with PBST and stained with phalloidin-rhodamine (1U per sample) and 300 nM DAPI (diluted in PBS) for 20 min. Samples were washed three times with PBS and the membranes were cut from the inserts and placed on glass slides. A drop of Vectashield mountant was placed on the surface of the tissue and a coverslip was sealed on top of the tissue layer using clear nail polish. Images were acquired using a Leica Dmi8 for standard fluorescence microscopy, while confocal images were acquired using a Zeiss LSM510.

### Quantitation of ciliation using immunofluorescence microscopy

To quantify ciliation, five independent locations on each β-tubulin-stained insert were acquired via a 20× objective. A fluorescence intensity threshold was applied in ImageJ such that only the ciliated regions were above the threshold. The area above the threshold was measured for each image and expressed as a percentage of the total area. A mean of three inserts was obtained for tissues derived from each of three independent animals.

### Sample preparation for histological and immunohistochemical analysis

ALI cultures were fixed in 4% (w/v) paraformaldehyde and stored in PBS. Cultures were processed by dehydrating through a series of increasing ethanol concentrations, cleared with xylene, infiltrated with paraffin wax and embedded in wax blocks. Sections (2.5μm in thickness) were cut using a Thermoshandon Finesse ME+ microtome and were stained with hematoxylin and eosin (H&E) or Periodic Acid Schiff (PAS) stain according to standard histological techniques. For immunohistochemistry (IHC), sections were subjected to antigen retrieval using a Menarini Access Retrieval Unit. Endogenous peroxidase was blocked using H_2_O_2_ in PBS. Sections were subsequently incubated with mouse anti-p63 antibody (Abcam #ab735) at a 1:30 dilution for 30 min followed by the application of an anti-mouse HRP-labelled polymer, before visualization with a REAL EnVision Peroxidase/DAB+ Detection System (Dako #K3468) according to manufacturer’s instructions. Samples were counterstained with Gill’s hematoxylin before dehydration, clearing and mounting in synthetic resin. Slides were visualized using a Leica DM2000 microscope.

### Sample preparation for scanning electron microscopy (SEM)

ALI cultures were fixed in 1.5% (v/v) glutaraldehyde in 0.1M sodium cacodylate for 1 h at 4°C. The apical and basal chambers were washed three times with 0.1M sodium cacodylate, five hundred microliters of 2% (w/v) osmium tertraoxide were added to the apical surface and the cells were incubated for 1 h at room temperature. Three 10 min washes were carried out with distilled water before staining with 0.5% (w/v) uranyl acetate for 1 h in the dark. The ALI cultures were washed twice with distilled water and dehydrated through increasing concentrations of ethanol. Samples were further dehydrated by incubation in hexamethyldisilizane before placing in a desiccator overnight. The membranes were cut from the inserts and mounted on aluminium SEM stubs and gold sputter-coated before analysing on a Jeol 6400 scanning electron microscope.

## Results

### General epithelial cell morphology and polarization

**Fig 1:**
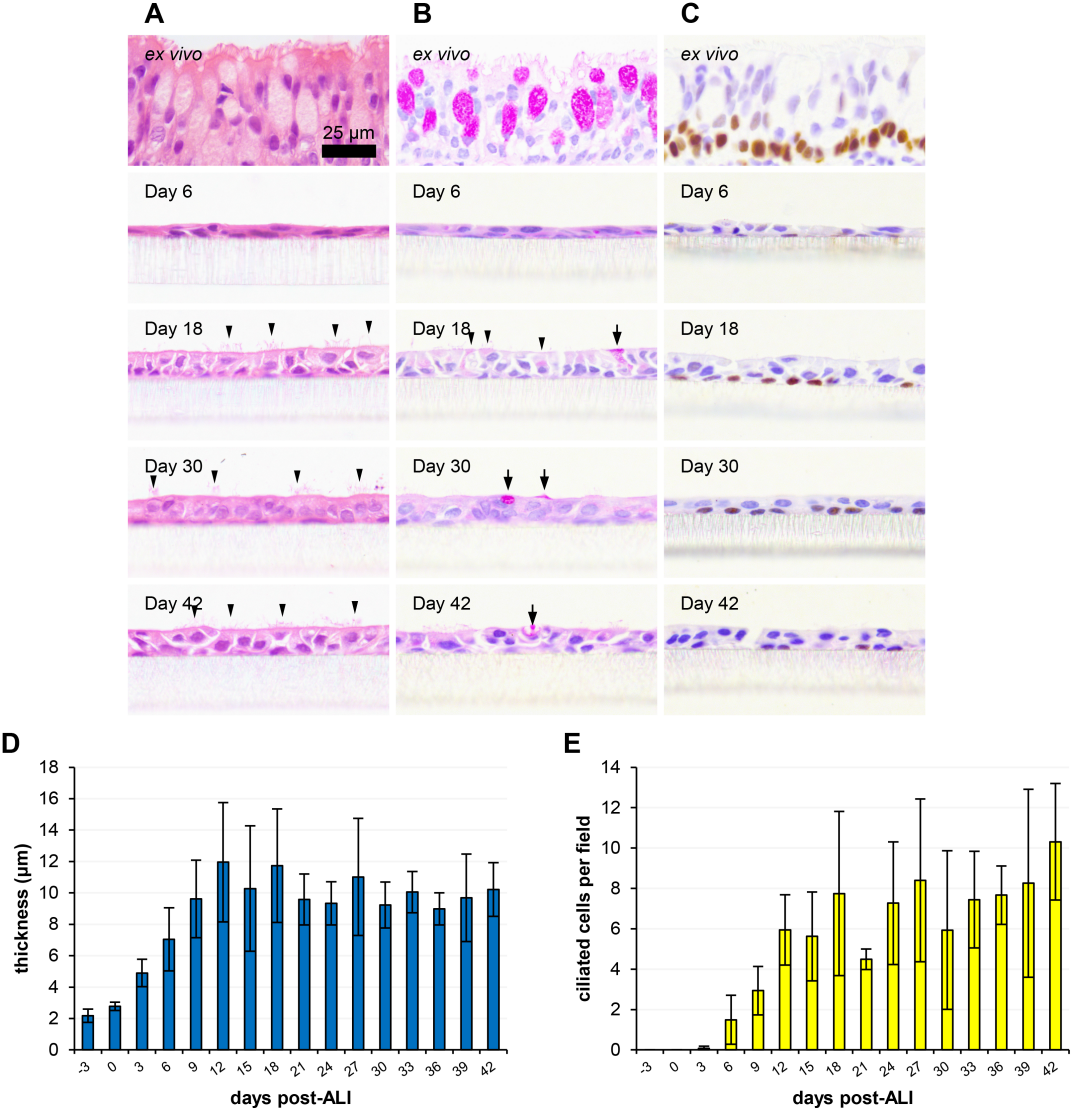
Histological assessment of ovine tracheal epithelial cell culture differentiation over time. Ovine tracheal epithelial cell cultures were grown at an ALI for the indicated number of days (relative to establishment of the ALI), fixed and paraffin embedded using standard histological techniques. Samples of *ex vivo* tissue were dissected from the center of each trachea prior to cell extraction, fixed, embedded and processed. Sections were taken, deparaffinized and stained as follows. (A) H&E staining of tissue layers at the indicated time points; selected ciliated cells are indicated by arrowheads. (B) PAS staining to detect mucus-containing/secreting cells (indicated by arrows and arrowheads). (C) Labelling of the transcription factor p63 to detect basal stem cells (positively labelled cells possess brown labelled nuclei). (D) Cell layer thickness was measured using ImageJ. Five images (400× magnification) were taken per insert with three points being measured per image. (E) The numbers of ciliated cells per field were counted from five images per insert. Three inserts were analysed per time point and the data represented is the mean +/- standard deviation from tissues derived from three independent animals (D and E). One-way ANOVA with post-test for linear trend was performed on data with significant (*P*<0.001) increasing trends being observed for both thickness (D) and ciliation (E).

Histological analysis allowed for overall assessment of the polarization and differentiation of the ovine ALI cultures in a temporal manner (Fig 1). Samples were analysed over a 45 day time period encompassing a single sample prior to establishment of the ALI (i.e. day -3) to 42 days post-ALI at 3 day intervals (Fig 1; S1–S4 Figs). This allowed for high resolution assessment of growth, proliferation and differentiation of the cell layer. General morphological observations and characterization of a variety of cell types including ciliated and goblet cells could be attained by standard H&E staining (Fig 1A), while PAS staining (Fig 1B) and p63 immunohistochemistry (Fig 1C) allowed for specific labelling of the mucus-producing and basal cell sub-populations, respectively. Transition from a squamous, sub-confluent, unpolarized epithelium to a well-differentiated ALI culture occurred between day -3 and day 21 (Fig 1D and 1E; S1 Fig). The epithelium was found to thicken following confluency, such that a mean thickness of 12 μm was reached by day 12 post-ALI (Fig 1D). The thickness of the epithelium was relatively stable for the remainder of the time-course with no significant increase/decrease in thickness being observed. While the ALI cultures were considerably thinner than the *ex vivo* tracheal epithelium, the pseudo-stratified morphology associated with these tissues was maintained–the tissue layer was two cells thick from day 12 post-ALI (S4A Fig) and the vast majority of cells maintained contact with the underlying membrane (Fig 1A, 1B and 1C). Ciliation and mucus production will be discussed in greater detail below. The basal progenitor cell marker p63 was detected at all time-points post-differentiation by IHC and was localized almost exclusively to basally-located cells with basal cell-like morphology (Fig 1C; S3 Fig). The squamous tissue layers observed prior to day 6 were lost during the antigen retrieval process and as such could not be analysed by IHC (S3 Fig). To assess de-differentiation and cell death, pyknotic and vacuolated cells were enumerated. Pyknosis or nuclear condensation is a feature of apoptosis, while vacuolation is often a feature of autophagy [46]. While a mean of 1.0 pyknotic and 0.37 vacuolated cells per field were identified between 21 and 42 days post-ALI (S4C and S4D Fig), the tissue layer appeared remarkably stable and a trend towards increased pyknosis and vacuolation at later time-points was not observed.

### Ciliation

**Fig 2:**
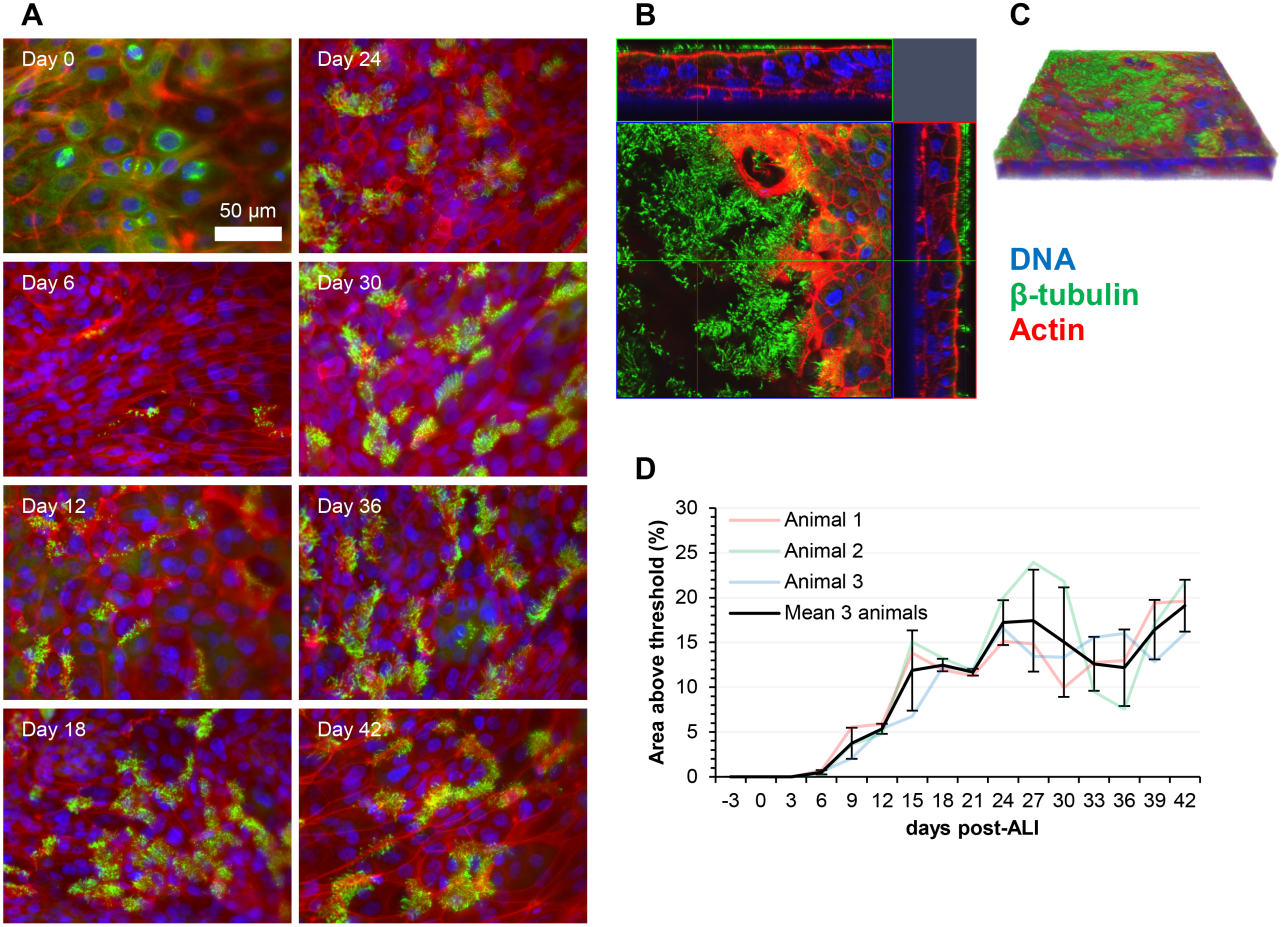
Ovine tracheal epithelial cell cultures display a time-dependent increase in apical surface ciliation. (A) Ovine tracheal epithelial cell cultures were grown at an ALI for the indicated number of days (relative to establishment of the ALI), fixed and immunostained using an anti-β tubulin antibody to detect cilia and rhodamine-phalloidin to stain the actin cytoskeleton. (B) Z-stack orthogonal representation of 21-day post-ALI tissue layer. (C) 3-dimensional representation of the Z-stack in panel B. (D) Ciliation was quantified by measuring the area above a manual fluorescence intensity threshold in ImageJ. For each time point, five regions on three independent cell cultures were measured. Results displayed are the mean +/- standard deviation from tissue layers derived from three animals.

**Fig 3:**
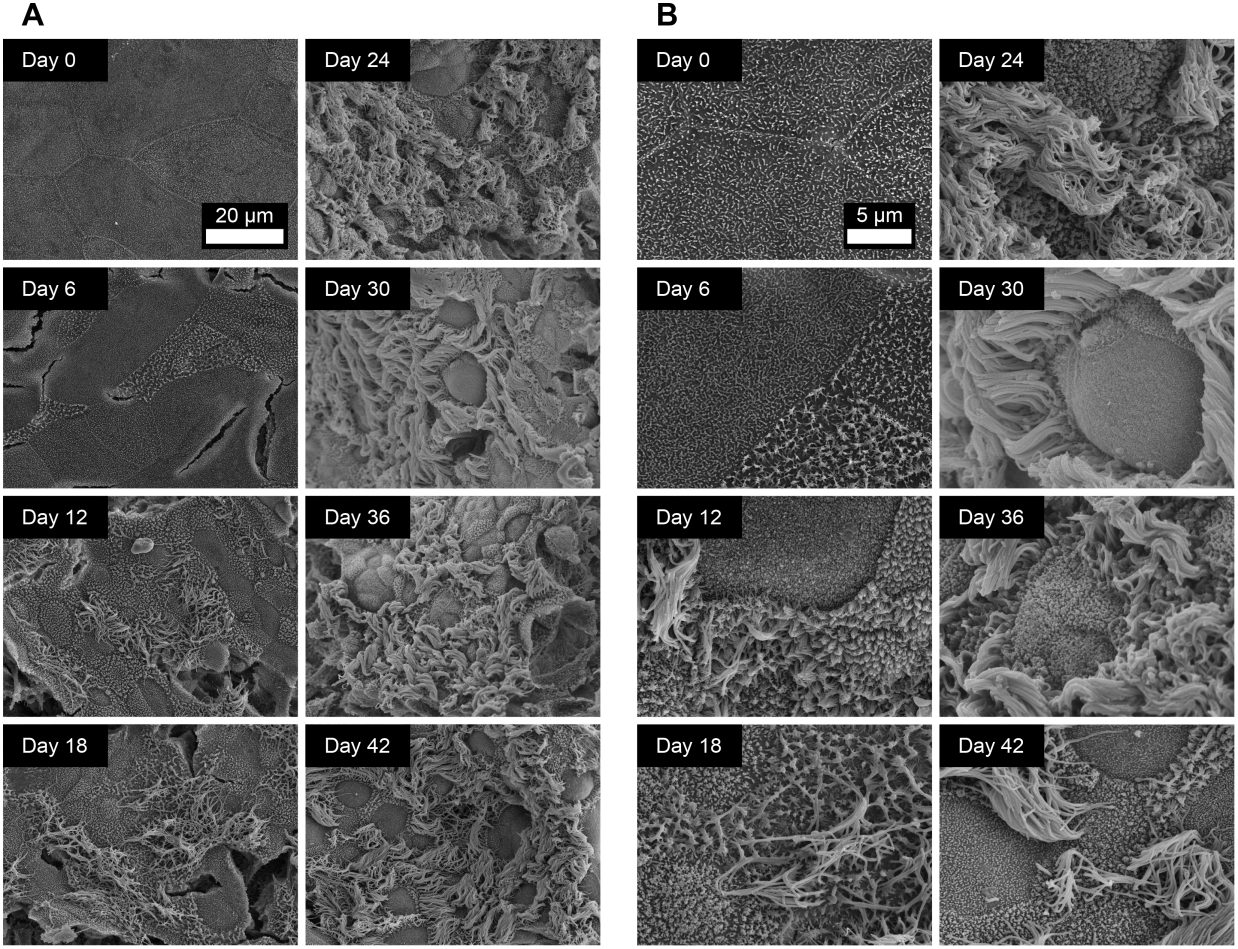
Ultrastructural analysis of the apical surface of ovine tracheal epithelial cell cultures by SEM. Ovine tracheal epithelial cell cultures were grown at an ALI for the indicated number of days (relative to establishment of the ALI), fixed and processed for SEM. (A) Images were taken at 1500× magnification. (B) Images were taken at 5000× magnification. Ciliated epithelial cells were observed from day 12 onwards.

Qualitative assessment of the temporal dynamics of ciliogenesis was revealed by histological analysis (Fig 1A) and immunofluorescent labelling of the cilia-associated protein β-tubulin (Fig 2; S5 Fig). As the epithelial cells proliferated and confluency was approached, the cell layer contained predominantly large, squamous epithelial cells with numerous cytoskeletal microtubules and microtubular mitotic spindles being visible (Fig 2A; S5 Fig). Apically-localized cilia were first observed at day 6 post-ALI. Cilial staining could be easily distinguished from cytoskeletal β-tubulin staining due to the relatively high intensity of labelling and its apical localization (Fig 2; S5 Fig). Quantitative measurement of ciliation was achieved both by counting ciliated cells in H&E-stained histological sections (Fig 1E) and by immunofluorescent staining (Fig 2D). Measurements using both methods showed good levels of correlation. Ciliation increased steadily from day 6 to day 24 (Figs 1A, 1E, 2A and 2D; S1 and S5 Figs), approaching mean ciliation levels of 7.47 ciliated cells per field (Fig 1E) and 15.2% of total area above fluorescence intensity threshold (Fig 2D) between days 21 and 42 post-ALI. Once maximum levels of ciliation were reached, no progressive decrease in ciliation was observed towards the later time-points of analysis. SEM analysis of the apical surface showed the emergence of short cilial outgrowths by day 6 (Fig 3; S6 and S7 Figs). The cilia increased in both length and abundance as the time-course proceeded with maximal levels being approached by day 24 (Fig 3; S6 and S7 Figs) in agreement with histological enumeration and immunofluorescent quantitation (Figs 1E and 2D). Importantly, no shortening or decreased abundance of cilia occurred by day 42 (Figs 1A, 1E, 2 and 3; S5, S6 and S7 Figs). Further detail on ultrastructural analysis will be provided below. Bright-field microscopy of low pore-density transparent inserts showed that the cilia were functional and capable of propelling mucus globules across the epithelial surface (S1 Movie).

#### Mucus production

**Fig 4:**
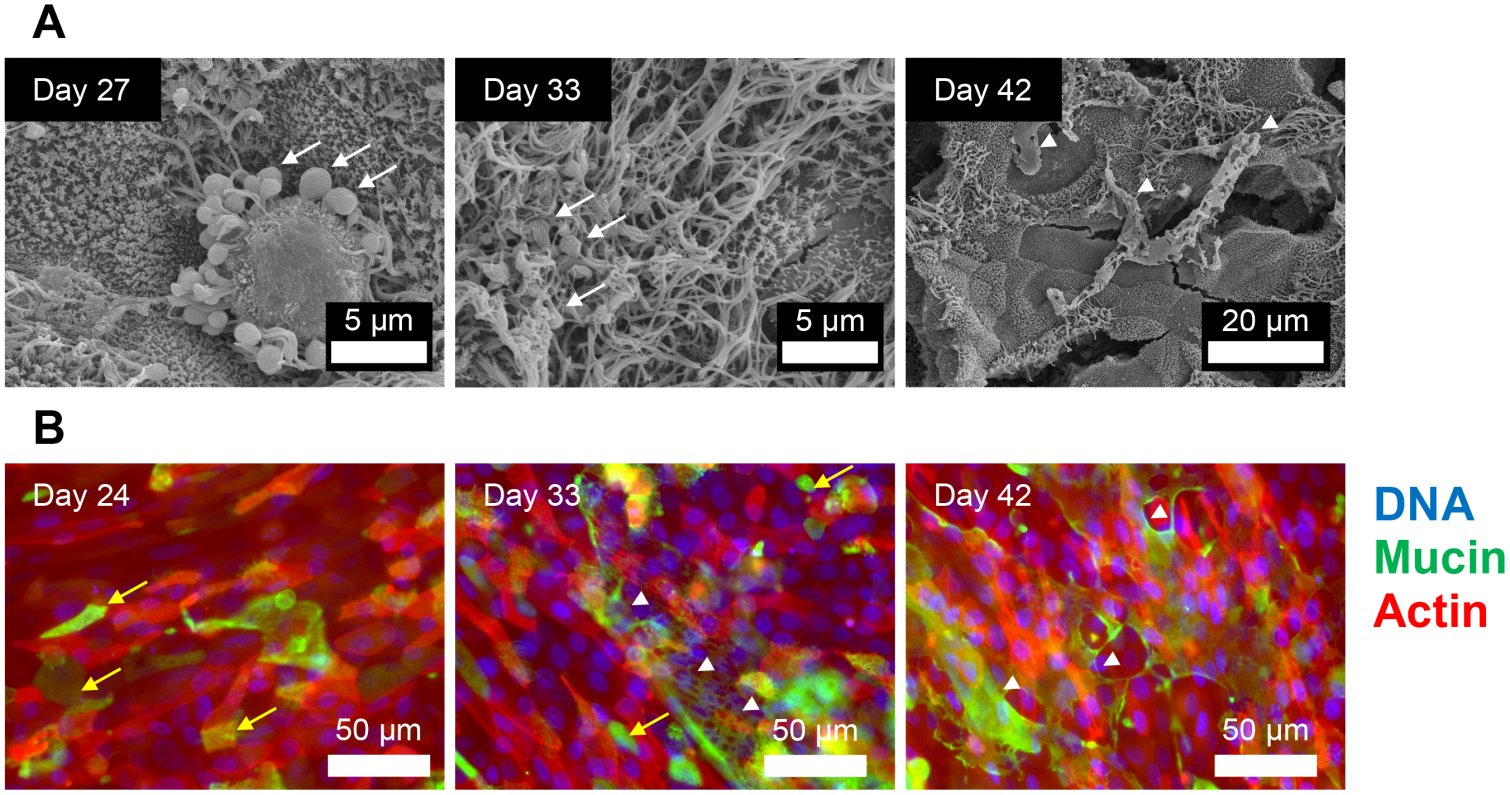
Mucus production by differentiated ovine tracheal epithelial cell cultures. (A) Ovine tracheal epithelial cell cultures were grown at ALI for the indicated number of days (relative to establishment of the ALI), fixed and processed for SEM. (B) Ovine tracheal epithelial cell cultures were grown for the indicated number of days, fixed and stained with jacalin-FITC (green), rhodamine-phalloidin (red) and DAPI (blue). Mucus globules are indicated by white arrows, carpets of amorphous mucus are indicated by white arrowheads and jacalin-labelled mucin-positive cells are indicated by yellow arrows.

In the respiratory tract mucus is produced by secretory goblet cells. We first attempted to quantitate goblet cells within the ALI cultures from H&E stained histological sections (S4B Fig). However, in many cases it was difficult to distinguish these cells as they failed to fully recapitulate the goblet cell morphology associated with the *ex vivo* tissue (Fig 1A and 1B). As such, we attempted to specifically label the polysaccharide-containing population of cells within the ALI cultures by PAS staining of histological sections (Fig 1B; S2 Fig). This analysis showed differential labelling of a subset of cells within the tissue layer from day 9 onwards (S2 Fig). Areas of faint positive staining were seen at earlier time-points, but since the cell layer was squamous prior to day 9, it was difficult to discern if these were true goblet-like cells. Some cells were PAS positive with relatively faint staining (arrowheads) while others showed very intense staining (arrows), similar to that observed in the *ex vivo* tissue (Fig 1B). Mucus production was also observed in isolated regions of the tissue layer by SEM (Fig 4A; S6 and S7 Figs) and by staining with jacalin-FITC lectin (Fig 4B) which has been shown to serve as a goblet cell marker in both native human airway tissue and well-differentiated human ALI cultures [47]. Mucus could be observed as web-like secretions, carpets of amorphous material or globules coating the cilia (Fig 4). In some cases, mucus was observed as globules being actively extruded from goblet cells (Fig 4A; S6 Fig [day 27]). Jacalin-staining allowed for detection of mucin-containing cells as early as day 0 (S8 Fig) indicating that the mucus-producing phenotype may develop independently of polarization, ciliation and epithelial thickening. The propulsion of clear globules of mucus by beating cilia on the surface of the cell layer is demonstrated in S1 Movie.

### Barrier function and junctional integrity

**Fig 5:**
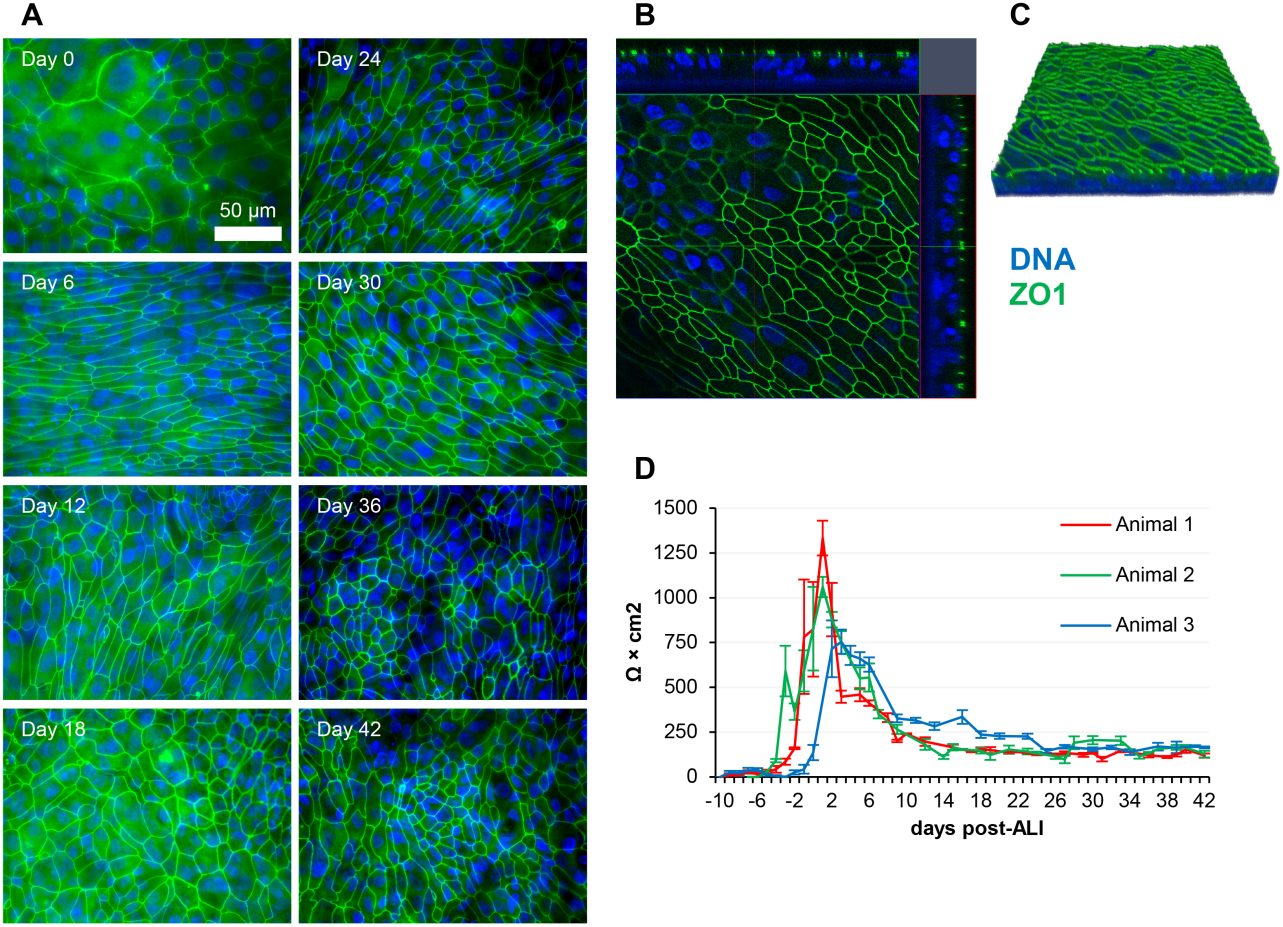
Ovine tracheal epithelial cell cultures display stable barrier function and junctional integrity. (A) Ovine tracheal epithelial cell cultures were grown at ALI for the indicated number of days (relative to establishment of the ALI) and tissue layers were fixed and immunostained using an anti-ZO1 antibody at the indicated time points (relative to establishment of the ALI). (B) Orthogonal representation of ALI culture at 24 days post-ALI. (C) 3-dimensional model of the Z-stack shown in panel B. (D) TEER measurements from four independent cell culture inserts at each time-point. Results for ALI cultures derived from three independent animals are shown (mean +/- standard deviation).

Tight junctions remained intact throughout the 45 days of analysis (three days prior to and 42 days post-ALI) as characterized by positive staining of the tight junctional protein ZO1 (Fig 5A; S9 Fig). However, the pattern of ZO1 varied temporally, as after confluency was reached, the cells became smaller and more numerous, and as such the number of junctions visible per field increased markedly. At later time-points it became difficult to focus on all of the tight junctions in a given field due to increased undulation of the apical surface. This undulation can be observed in the confocal Z-stacks (Figs 2B, 2C, 5B and 5C). Approximately 10 days of submerged growth was required for confluency to be achieved and a subsequent increase in TEER to be detected. The TEER reached its highest point during the early squamous phase of growth, with a mean peak value of 1049 Ω × cm^2^ being observed between day 1 and day 3 post-ALI (Fig 5D). A decrease in TEER occurred after this peak value was reached at day 1 or day 3 post-ALI (depending on the animal being analysed). However, this decrease was also found to occur in continuously submerged cells (data not shown) and, as such, this process cannot be attributed directly to establishment of the ALI. Interestingly, although TEER decreased as differentiation proceeded, junctional staining was not affected. TEER stabilized at ~200 Ω × cm^2^ and, importantly, the epithelial barrier remained intact, with no leakage of basal media being detected at later time-points.

### Ultrastructural features

High resolution assessment of differentiation of the epithelial surface was achieved by SEM. At days -3, 0 and 3 post-ALI, the cell layer was found to be squamous, completely devoid of cilia and quite uniform with microvilli protruding from the majority of cells within the culture (Fig 3; S6 and S7 Figs). As the cell layer thickened, cracking was observed in many areas. This was not observed in early squamous-like tissue and was likely an effect of tissue shrinkage during SEM sample preparation. At day 6, the cell surface features became more pronounced and short cilial outgrowths were observed in isolated regions. As differentiation proceeded (day 6 to day 24 post-ALI) these became more numerous and the cilia increased in length and a maximum apparent level of ciliation was observed at day 24. Between days 24 and 42 post-ALI, little difference was seen in the topography of the epithelial surface. Differentiated ALI cultures possessed ciliated cells with typical morphology, microvillous epithelial cells (possibly brush cells) and some raised epithelial cells with relatively short microvilli which may represent the mucus-producing goblet cell sub-population. Globules of mucus were occasionally observed as being actively secreted from such cells (Fig 4A) supporting the assumption that this cellular morphology is consistent with mucus production.

## Discussion

Respiratory tract infections represent one of the primary causes of mortality in ruminant livestock [29, 48, 49]. Detailed characterization of the bacteria and viruses responsible for such infections has been hampered by the lack of suitable infection models. In this study, we describe a well-differentiated organotypic model of the ovine tracheal epithelium which was obtained by culturing tracheal epithelial cells on semi-permeable membranes at an ALI. Airway epithelial cells grown under submerged conditions fail to develop the expression profile or the full complement of cell types present *in vivo* [23, 50]. Respiratory pathogens often target a subset of differentiated cells in the airway epithelium and as such, a fully differentiated model is required in order for all aspects of pathogenesis to be considered [27, 44, 51, 52]. Airway epithelial cells have also been shown to display altered inflammatory responses when differentiated for 3, 10 and 21 days at ALI [53]. Two similar studies described in excellent detail the proliferation, polarization, and cellular differentiation of human airway ALI cultures [21, 22], with a window between day 24 and 33 post-ALI where the model was fully differentiated and suitable for experimentation [21]. In both cases, some features of de-differentiation were observed after 21 days post-ALI. For these reasons, we carried out a detailed temporal analysis of differentiation over an extended time-period, so that comprehensive assessments could be made regarding the optimum time-points (with respect to differentiation) for conducting infection experiments.

Vertebrates have evolved a so-called “mucociliary escalator” phenotype within airway epithelial tissues in order to maintain a healthy environment in the deeper regions of the respiratory tract where gaseous exchange occurs [54]. This feature involves entrapment of particles within mucus globules and subsequent propulsion out of the airway, where they can be swallowed and passed into the gastro-intestinal tract. In order to be considered truly organotypic, it was essential that our model possessed both actively beating cilia and mucus production. We were able to detect ciliation via histology, immunofluorescence and SEM as early as day 6, with an increase in abundance of ciliated cells being observed up to day 24. In particular, immunofluorescent labelling of β-tubulin allowed for a high level of sensitivity and allowed for quantitative assessment of ciliation. A temporal analysis of primary human bronchial epithelial cells demonstrated ciliation by day 15 with full differentiation being reached by day 24 [21]. Functionally beating cilia have previously been observed within ovine tracheal ALI cultures, although production/propulsion of mucus droplets was not described [39]. We have demonstrated that the cilia produced in our model are capable of driving the movement of mucus globules across the epithelial surface in a similar manner to that seen in the mucociliary escalator *in vivo* (S1 Movie). While mucociliary functionality is typically thought of as being a central innate defense mechanism which inhibits colonization by pathogens, ciliated cells have also been shown to act as a target for adhesion with some species of viruses and bacteria [44, 51, 52, 55]. Studies on the modulation of ciliary beat frequency and co-ordination of cilial beating in response to drugs, pollutants and infectious agents have also yielded important insights into diverse pathologies [56, 57]. This model provides an excellent platform for further studies in such areas.

The junctional complexes of the airway epithelium contribute greatly to overall barrier function and resistance to infection in the respiratory tract [58–60]. Once confluency was reached in the submerged growth phase, TEER rapidly increased to 1049 Ω × cm^2^ and subsequently decreased to a stable 200 Ω × cm^2^ after establishment of ALI. Our model formed an electrically tight barrier and stable TEER readings of ~200 Ω × cm^2^ were observed throughout the differentiated phase of growth (days 21 to 42). Similar trends in TEER have been observed in porcine and equine airway ALI cultures [61, 62]. TEER values for Calu-3 airway cells have been shown to be higher when cultured under submerged conditions [63]. Importantly, staining of the tight junctional protein ZO1 was also maintained at a stable and consistent level throughout the time-course, with the exception of day -3 (3 days prior to ALI establishment) when the cell layer was sub-confluent. A number of bacterial and viral pathogens have been shown to be capable of disrupting the epithelial barrier *in vitro* [64–66]. The application of our model for investigating airway barrier disruption by ruminant pathogens will reveal new insights into tissue damage and pathogen dissemination during pneumonic infections.

PAS or alcian blue staining is routinely used to detect mucus-producing cells in histological sections. It has previously been shown that PAS-positive mucus-producing cells begin to appear in human airway ALI cell layers from approximately 6 days post-ALI and that these increase in abundance and intensity of staining up to day 21 [22]. An association between detection of PAS-positive cells and increasing transcription of MUC5AC and MUC5B (the two major secretory mucins) by RT-PCR within this time-frame was also observed [22]. We observed a similar trend with respect to the development of PAS-positive cells and demonstrated that this feature is stable during the 45 days of analysis. There was also a correlation between the detection of mucus-producing cells by PAS staining and the appearance of goblet-like cells on the apical surface as observed by SEM. When visualized by SEM, goblet cells have been described as having a swollen appearance with microvilli being more numerous around the periphery of the cell [67]. We were able to visualize mucus globules being actively extruded form such cells (Fig 4A; S6 Fig [day 27]) and globules of similar appearance were frequently found entangled in or underneath the apical cilia (Fig 4A [day 33]). We attempted to label goblet cells using two distinct anti-human MUC5AC antibodies by IHC and immunofluorescence. However, the antibodies failed to detect goblet cells in the sheep airway model (data not shown). The fact that sheep MUC5AC possesses 74% identity across 84% of the amino acid sequence of human MUC5AC and the fact that these proteins are heavily post-translationally modified (glycosylated) may explain the lack of cross-reactivity with these antibodies. Jacalin recognizes a mature O-glycosylated α-GalNac-linked form of Muc5AC [68] and has been successfully used to label goblet cells [47]. We identified a subset of jacalin-positive cells by immunofluorescence and also observed some sheet-like deposits of mucus (Fig 4B). This confirmed our findings from PAS staining of histological sections and SEM. Many bacterial species are capable of degrading the mucus layer via specific mucinases and this plays a key role in epithelial colonization [69, 70]. Respiratory conditions in humans such as asthma and cystic fibrosis are thought to be exacerbated by the fact that bacterial species such as *Mycoplasma pneumoniae* induce increased mucin expression [71]. *Pseudomonas aeruginosa* binds to airway mucins and this is thought to play a major role in airway colonization in cystic fibrosis [72]. These relationships are not fully understood in the case of ruminant respiratory disease. Therefore, the mucus-producing phenotype of our model represents an attractive avenue for such investigations.

Basal cells constitute the main progenitor cell type in the airway epithelium and develop into differentiated epithelial cells during post-natal growth [5]. Basal cells continue to act as progenitor cells in the adult airway epithelium facilitating repair of damaged tissues [5, 73]. We have demonstrated that our model possesses p63-positive basal cells throughout the time-period analysed. A number of important respiratory viruses target the basal cell sub-population during bovine and human infections [51, 74, 75]. Such tropisms have not been investigated for the major bacterial or viral pathogens of the sheep respiratory tract. Our model will allow for interesting insights into potential pathogen-basal cell interactions.

An abundant, non-ciliated epithelial cell type was identified by SEM analysis, the apical surfaces of which displayed numerous microvilli. Pulmonary microvillous epithelial cells, also termed brush cells, are characterized as having a pear-shaped morphology in cross-section and longer microvilli than those of goblet cells [67, 76]. Until recently, the function of these cells remained elusive, although they were thought to have a secretory function due to their possession of large numbers of cytoplasmic vesicles [76]. Recent work identified that brush cells possess a sensory network, similar to that involved in bitter taste sensation, which can regulate the frequency of breathing [8]. These cells are also capable of detecting bacterial quorum-sensing N-acyl homo-serine lactones [77]. The presence of these cells in the differentiated ALI cultures further enhances the representative nature of the model and may indicate sensory capabilities which would be important in the *in vitro* characterization of epithelial infection.

While we have shown that good levels of differentiation can be achieved in the ovine ALI culture system, we have not provided mechanistic insight into the regulation of these processes at the transcriptional level. This could be facilitated by extracting and purifying mRNA at a variety of time-points in order to determine the levels of expression of differentiation-related targets using RNAseq or qPCR. Although the transcriptional profile of differentiated primary ALI cultures can closely mimic that of the airway epithelium *in vivo* [23, 78] a poor correlation between mRNA levels and observable levels of mature protein by immunolabelling has also been described [79]. Furthermore, miRNA levels have been shown to vary considerably between freshly isolated airway epithelial cells and differentiated ALI cultures [80]. As such, we chose to assess levels of differentiation using antibody labelling, thereby avoiding any inherent analytical complications due to post-transcriptional regulation/modification, while also allowing confirmation of correct sub-cellular localization.

One of the most promising features of the model described in this study is the long-term stability of the differentiated cell layer. Pyknosis or nuclear condensation is a feature of apoptosis, while vacuolation is often a feature of autophagy [46]. While both features were observed at various time-points post-differentiation, a trend towards increased features of cell death was not observed at later time-points (S4C and S4D Fig). A number of previous time-course analyses have detailed a limited lifespan for ALI cultures with deterioration as evidenced by decreased ciliation and the formation of pores/vacuoles in the cell layer [21, 81]. Indeed, a number of primary research articles and reviews have stated that the limited lifespan of primary airway epithelial cells represents the most prominent drawback to their use [21, 79, 82, 83]. Although we have not addressed the issue of repeated subculture and expansion in the present study, from a single expansion of cells derived from a single trachea, we routinely acquired sufficient numbers of cells for seeding 200 inserts. Once differentiated, these cultures were stable for the entire 42 days of analysis. We believe that this would allow sufficient time for long-term experimentation. One particularly attractive avenue of research, given the multi-factorial nature of ruminant respiratory disease, would be to investigate the involvement of viruses in pre-disposition of epithelia to infection by bacterial members of the respiratory disease complex (RDC). Recently, a study involving viral infection of bovine ALI cultures [51] and another involving *in vivo* cattle infection with bacterial and viral agents of the RDC [84] described the variable and exacerbatory roles played by each agent in causing progression of the disease. Similar exacerbation of pneumonic pathology has been seen with *in vivo* pre-exposure of sheep to *Mycoplasma ovipneumoniae*, followed by infection with the most common bacterial cause of respiratory disease in sheep–*M*. *haemolytica* [32]. *Bibersteinia trehalosi*, respiratory syncytial virus, and parainfluenza-3 virus have also been shown to play synergistic roles with *M*. *haemolytica* in inducing pneumonia in sheep by *in vivo* infection [33]. Our model would allow for analysis of this complex process without a need for expensive and ethically questionable *in vivo* infection.

The limitations of primary cell culture systems have been well documented and include cost, finite lifespan, limited cell numbers and inter-donor variability [21, 79, 82, 83, 85]. Our model addresses the issues of cost and limited resource/cell numbers as the tissues are easily obtainable at a low cost from meat production facilities. Little variation was observed in terms of overall differentiation of tracheal epithelial cells derived from three animals, demonstrating that good levels of differentiation can be obtained consistently. We identified a wide window between days 21 and 42 post-ALI in which the model is well-differentiated and suitable for experimentation. The model is highly representative of the airway epithelium *in vivo*; it possesses all of the major cell types found within airway epithelia including basal progenitor cells, ciliated cells and mucus-producing goblet cells. Importantly, the model is highly stable with good levels of differentiation being observed over a three-week period. As such, it is suitable for long term experiments in numerous diverse applications.

## Supporting information

S1 Fig Temporal progression of growth and differentiation of ovine tracheal epithelial cell cultures. Ovine tracheal epithelial cells were cultured to confluency and an ALI was established on day 0. Samples were taken 3 days prior to establishing the ALI and at 3 day intervals until day 42 post-ALI. At each time point samples were fixed, processed for histological analysis and stained with H&E.
(TIF)

S2 Fig Temporal analysis of mucin production in ovine tracheal epithelial cell cultures by PAS staining. Ovine tracheal epithelial cells were cultured to confluency and an ALI was established on day 0. Samples were taken 3 days prior to establishing the ALI and at 3 day intervals until day 42 post-ALI. At each time point samples were fixed, processed for histological analysis and stained with PAS stain.
(TIF)

S3 Fig Immunohistochemistry reveals the presence of basal stem cells throughout the growth and differentiation of ovine tracheal epithelial cell cultures. Ovine tracheal epithelial cells were cultured to confluency and an ALI was established on day 0. Samples were taken 3 days prior to establishing the ALI and at 3 day intervals until day 42 post-ALI. At each time point samples were fixed, processed for histological analysis, subjected to antigen retrieval and labelled with an anti-p63 antibody followed by counterstaining with haematoxylin. P63-positive basal stem cells are indicated by possession of brown nuclei. For days -3, 0 and 3 the tissue layers were too thin to be recovered following antigen retrieval.
(TIF)

S4 Fig Assessment of differentiation- and deterioration-related traits from histological sections. Five images (400× magnification) were taken per insert and three inserts were analysed per time-point. The data represents the mean plus/minus standard deviation from tissues derived from three independent animals. (A) Cell layer thickness as determined by counting the number of cells thick from three locations in each image. (B) Number of goblet cells per field. Inset is an example of a typical goblet cell. (C) Number of cells with pyknotic nuclei per field. Inset is an example of a pyknotic cell. (C) Number of vacuoles per field. Inset is an example of a vacuolated cell.
(TIF)

S5 Fig Ovine tracheal epithelial cell cultures produce ciliated epithelial cells which are stable up to day 42 post-ALI. Ovine tracheal epithelial cell cultures were grown at an ALI for the indicated number of days, fixed and immunostained using an anti-β tubulin antibody to detect cilia (green) and rhodamine-phalloidin to stain the actin cytoskeleton (red). DAPI was used to stain nuclear DNA (blue). Mitotic spindles are indicated by arrowheads, selected cells exhibiting pronounced labelling of cytoskeletal microtubules are indicated by arrows.
(TIF)

S6 Fig Ultrastructural analysis of ovine tracheal epithelial cell culture differentiation over time. Ovine tracheal epithelial cell cultures were grown on cell culture inserts at an ALI and tissue layers at the indicated time points were fixed, processed and analysed by SEM. *Ex vivo* tissues were dissected prior to cell extraction and were also fixed, processed and analysed by SEM.
(TIF)

S7 Fig Ultrastructural analysis of ovine tracheal epithelial cell culture differentiation over time. Ovine tracheal epithelial cell cultures were grown on cell culture inserts at an ALI and tissue layers at the indicated time points were fixed, processed and analysed by SEM. *Ex vivo* tissues were dissected prior to cell extraction and were also fixed, processed and analysed by SEM.
(TIF)

S8 Fig Ovine tracheal epithelial cell cultures develop mucus-producing cells which can be detected by jacalin-FITC lectin. Ovine tracheal epithelial cell cultures were grown at an ALI for the indicated number of days (relative to establishment of the ALI), fixed and stained using jacalin-FITC to detect mucins (green) and rhodamine-phalloidin to stain the actin cytoskeleton (red). DAPI was used to stain nuclear DNA (blue).
(TIF)

S9 Fig Ovine tracheal epithelial cell cultures produce an epithelial barrier with stable tight junctions. Ovine tracheal epithelial cell cultures were grown at an ALI for the indicated number of days (relative to establishment of the ALI), fixed and immunostained using an anti-ZO1 antibody (green). DAPI was used to stain nuclear DNA (blue).
(TIF)

S1 Movie Differentiated ovine tracheal epithelial cell cultures possess actively beating cilia which are capable of propelling mucus globules. Movie was captured from day 14 post-ALI ovine tracheal epithelial cell culture using a Leica Dmi1 inverted microscope.
(MP4)

S1 File Underlying data. (XLSX)
