# Supplementary figures and images for: Temporal dynamics of ovine airway epithelial cell differentiation at an air-liquid interface
Source: PLoS One. 2017 Jul 26;12(7):e0181583. doi: 10.1371/journal.pone.0181583 (PMC5529025; doi:10.1371/journal.pone.0181583)

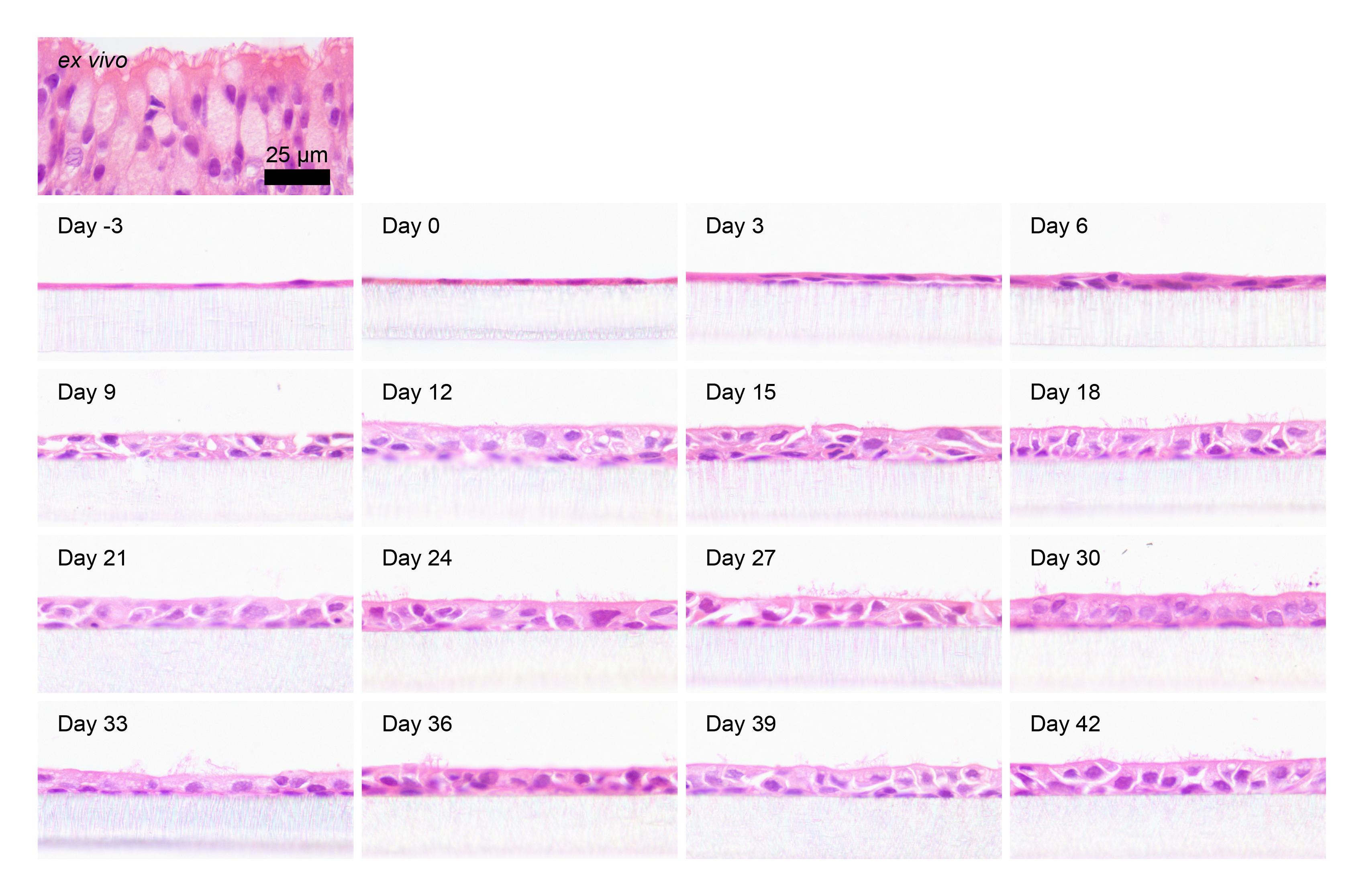

Supplement: S1 Fig — Ovine tracheal epithelial cells were cultured to confluency and an ALI was established on day 0. Samples were taken 3 days prior to establishing the ALI and at 3 day intervals until day 42 post-ALI. At each time point samples were fixed, processed for histological analysis and stained with H&E. (TIF) [file pone.0181583.s001.tif]

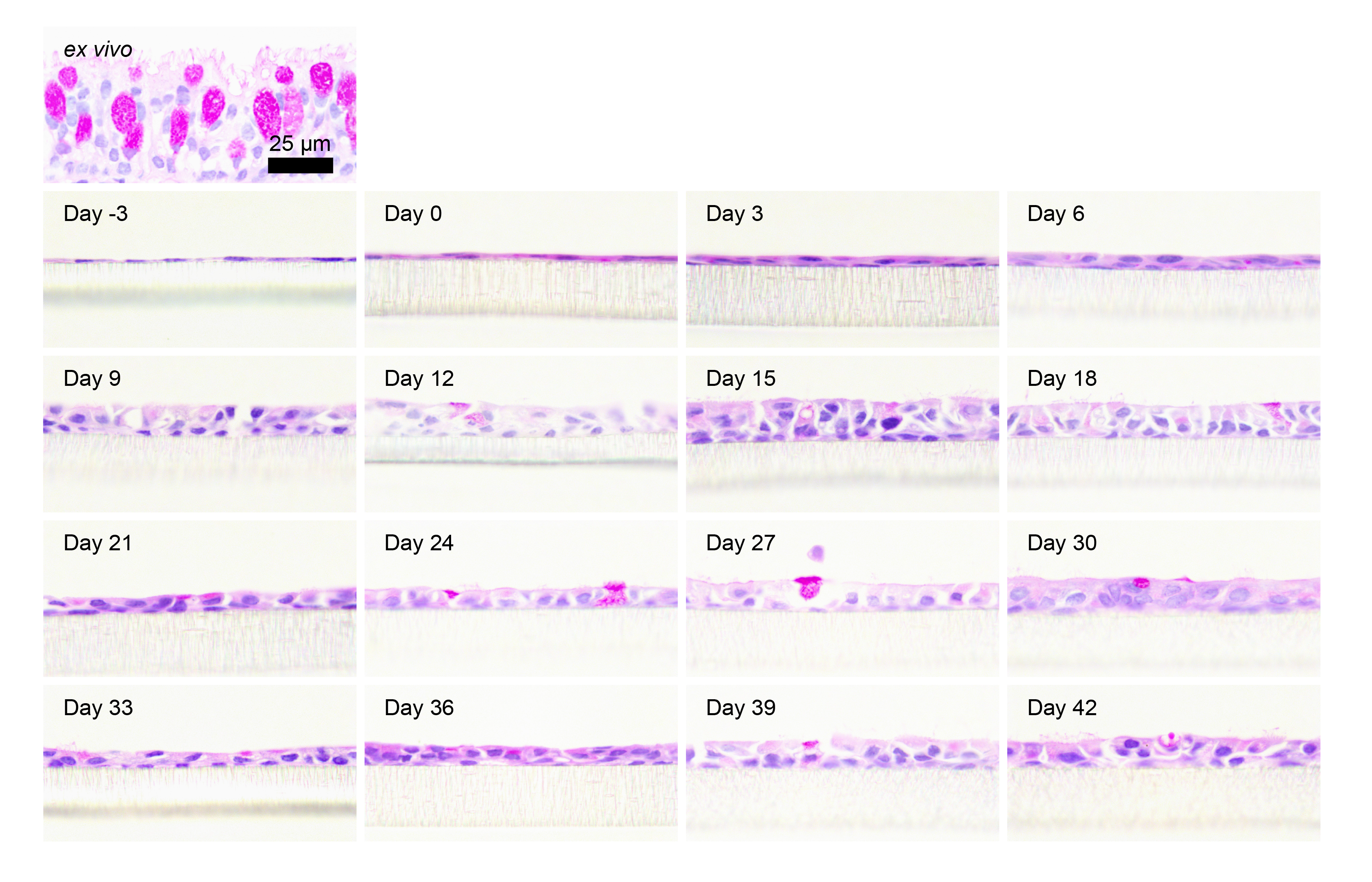

Supplement: S2 Fig — Ovine tracheal epithelial cells were cultured to confluency and an ALI was established on day 0. Samples were taken 3 days prior to establishing the ALI and at 3 day intervals until day 42 post-ALI. At each time point samples were fixed, processed for histological analysis and stained with PAS stain. (TIF) [file pone.0181583.s002.tif]

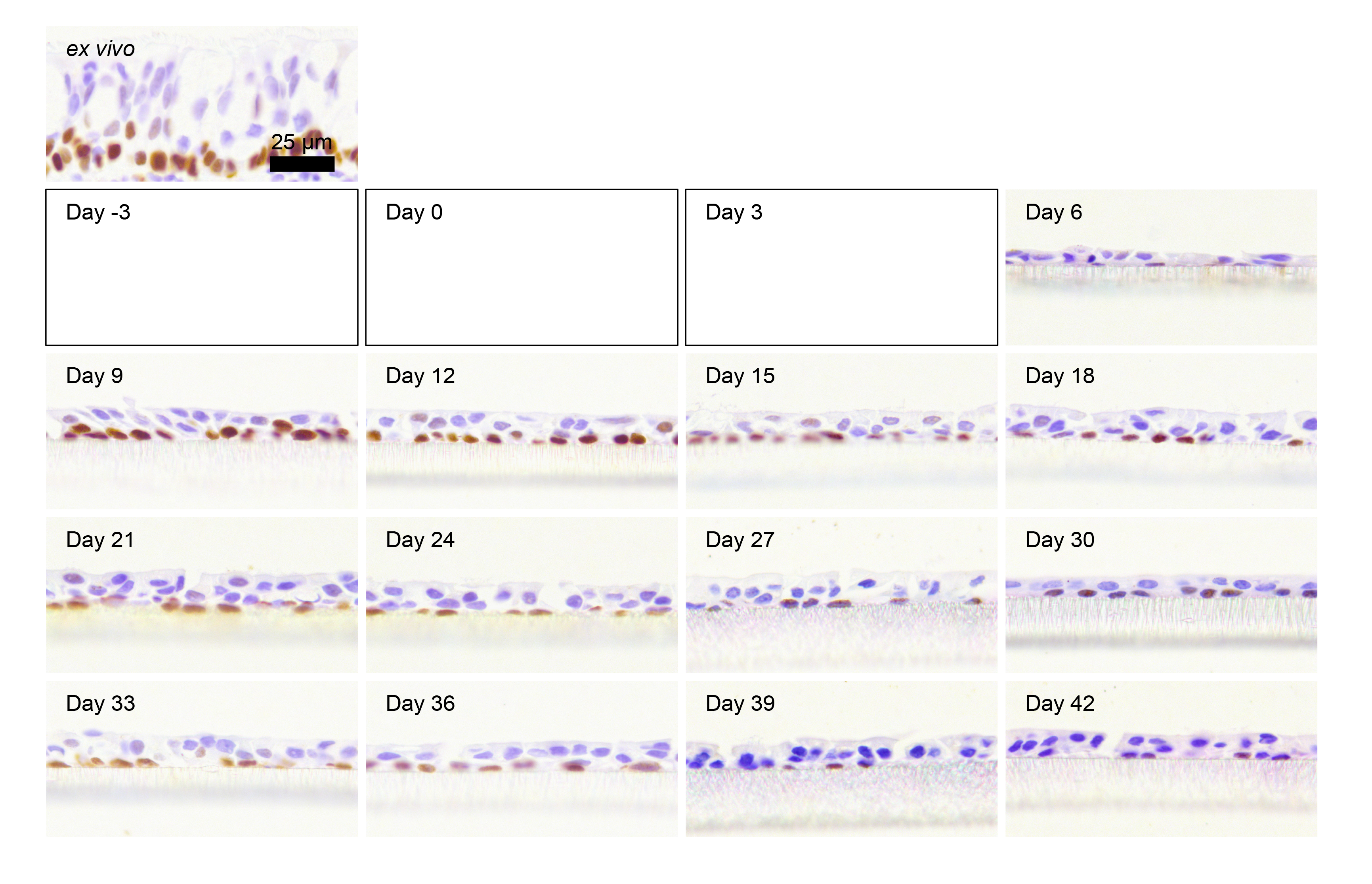

Supplement: S3 Fig — Ovine tracheal epithelial cells were cultured to confluency and an ALI was established on day 0. Samples were taken 3 days prior to establishing the ALI and at 3 day intervals until day 42 post-ALI. At each time point samples were fixed, processed for histological analysis, subjected to antigen retrieval and labelled with an anti-p63 antibody followed by counterstaining with haematoxylin. P63-positive basal stem cells are indicated by possession of brown nuclei. For days -3, 0 and 3 the tissue layers were too thin to be recovered following antigen retrieval. (TIF) [file pone.0181583.s003.tif]

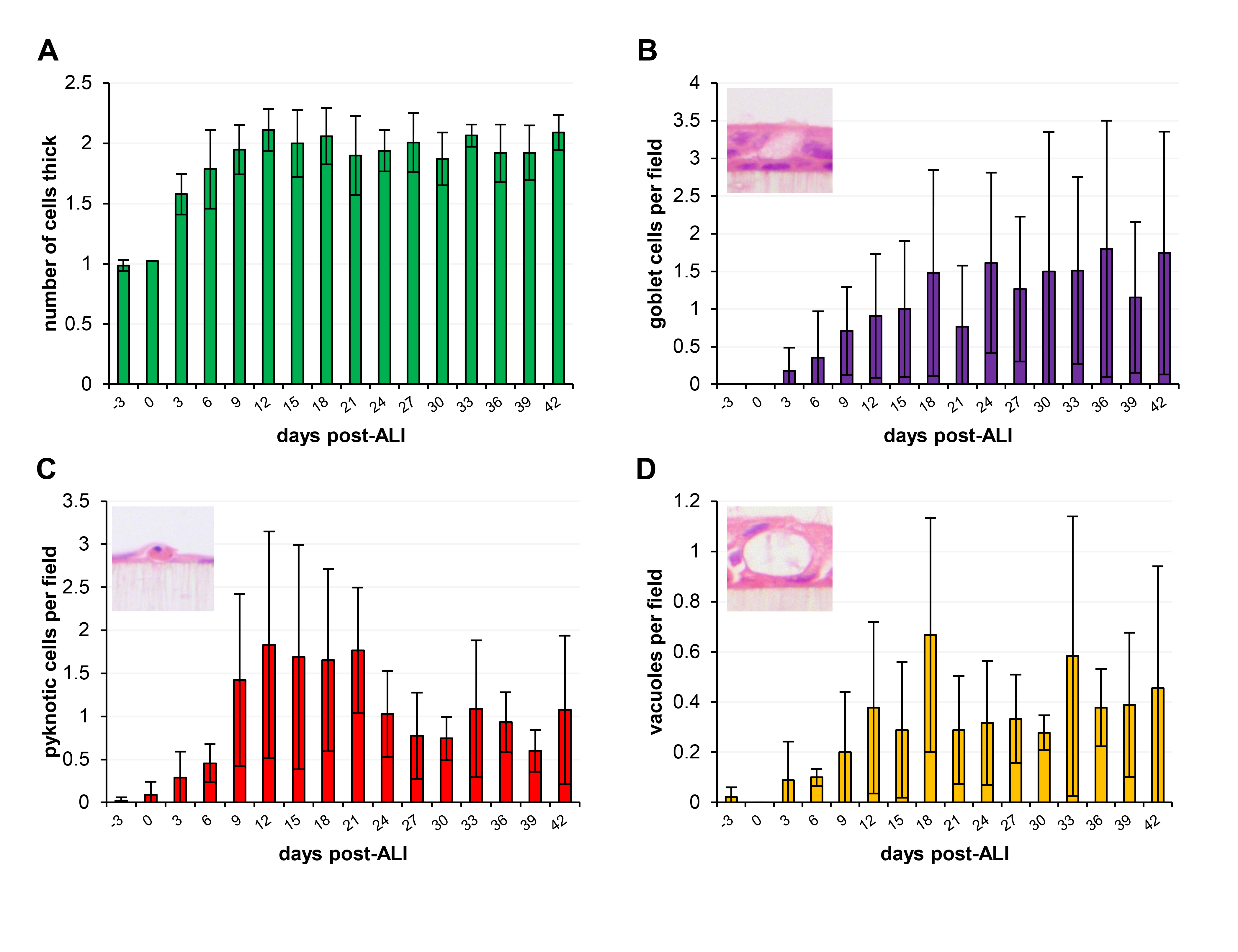

Supplement: S4 Fig — Five images (400× magnification) were taken per insert and three inserts were analysed per time-point. The data represents the mean plus/minus standard deviation from tissues derived from three independent animals. (A) Cell layer thickness as determined by counting the number of cells thick from three locations in each image. (B) Number of goblet cells per field. Inset is an example of a typical goblet cell. (C) Number of cells with pyknotic nuclei per field. Inset is an example of a pyknotic cell. (C) Number of vacuoles per field. Inset is an example of a vacuolated cell. (TIF) [file pone.0181583.s004.tif]

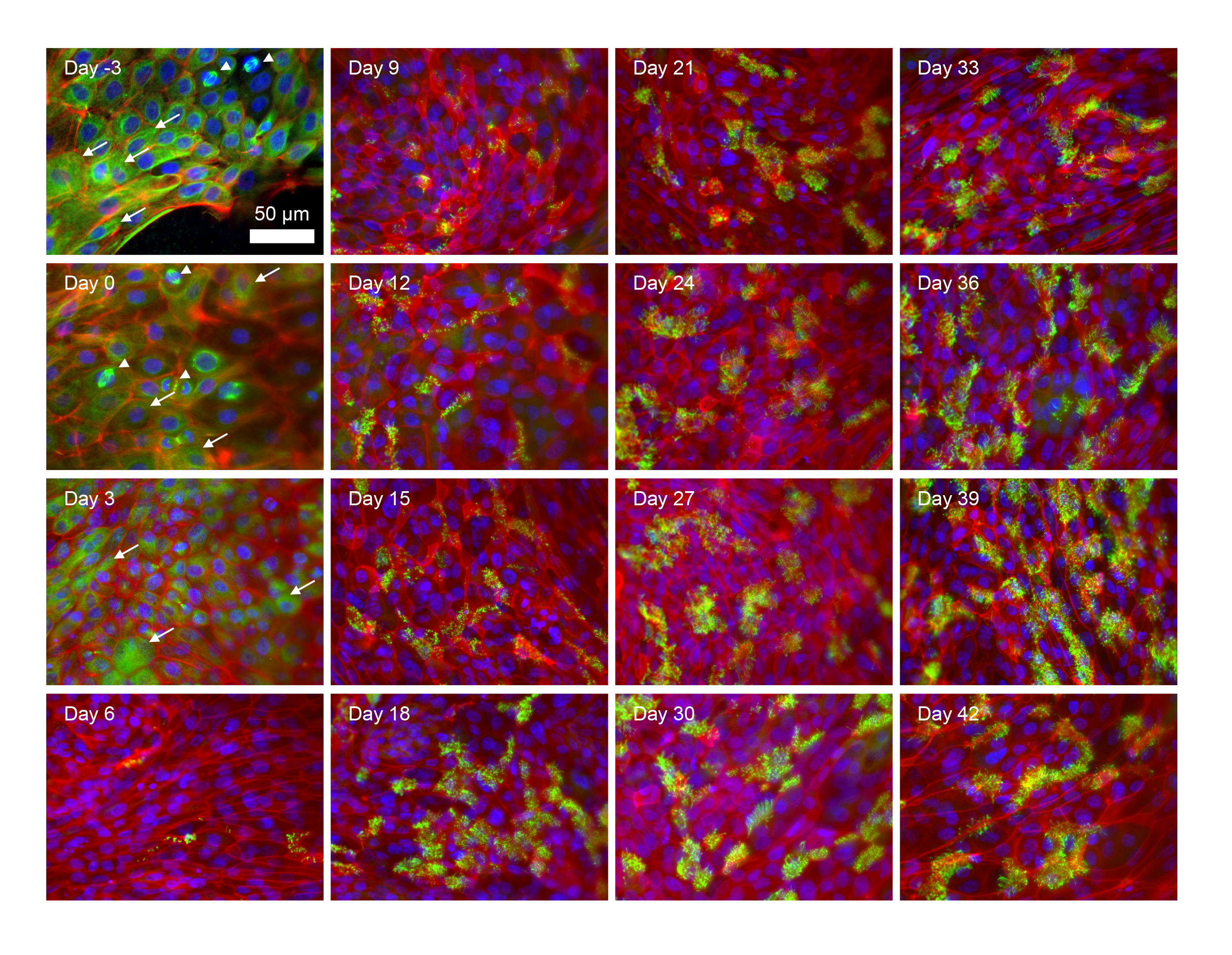

Supplement: S5 Fig — Ovine tracheal epithelial cell cultures were grown at an ALI for the indicated number of days, fixed and immunostained using an anti-β tubulin antibody to detect cilia (green) and rhodamine-phalloidin to stain the actin cytoskeleton (red). DAPI was used to stain nuclear DNA (blue). Mitotic spindles are indicated by arrowheads, selected cells exhibiting pronounced labelling of cytoskeletal microtubules are indicated by arrows. (TIF) [file pone.0181583.s005.tif]

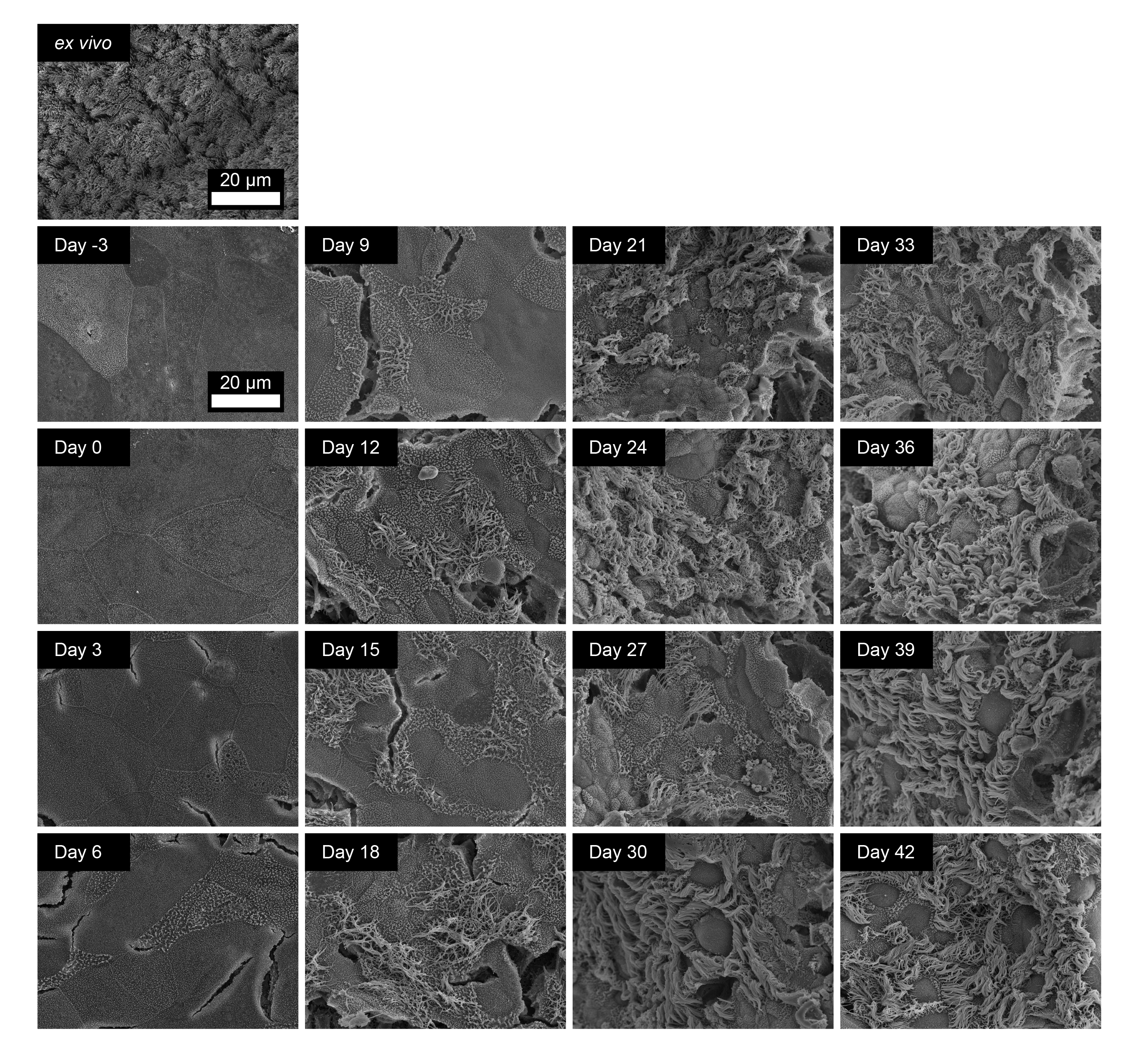

Supplement: S6 Fig — Ovine tracheal epithelial cell cultures were grown on cell culture inserts at an ALI and tissue layers at the indicated time points were fixed, processed and analysed by SEM. Ex vivo tissues were dissected prior to cell extraction and were also fixed, processed and analysed by SEM. (TIF) [file pone.0181583.s006.tif]

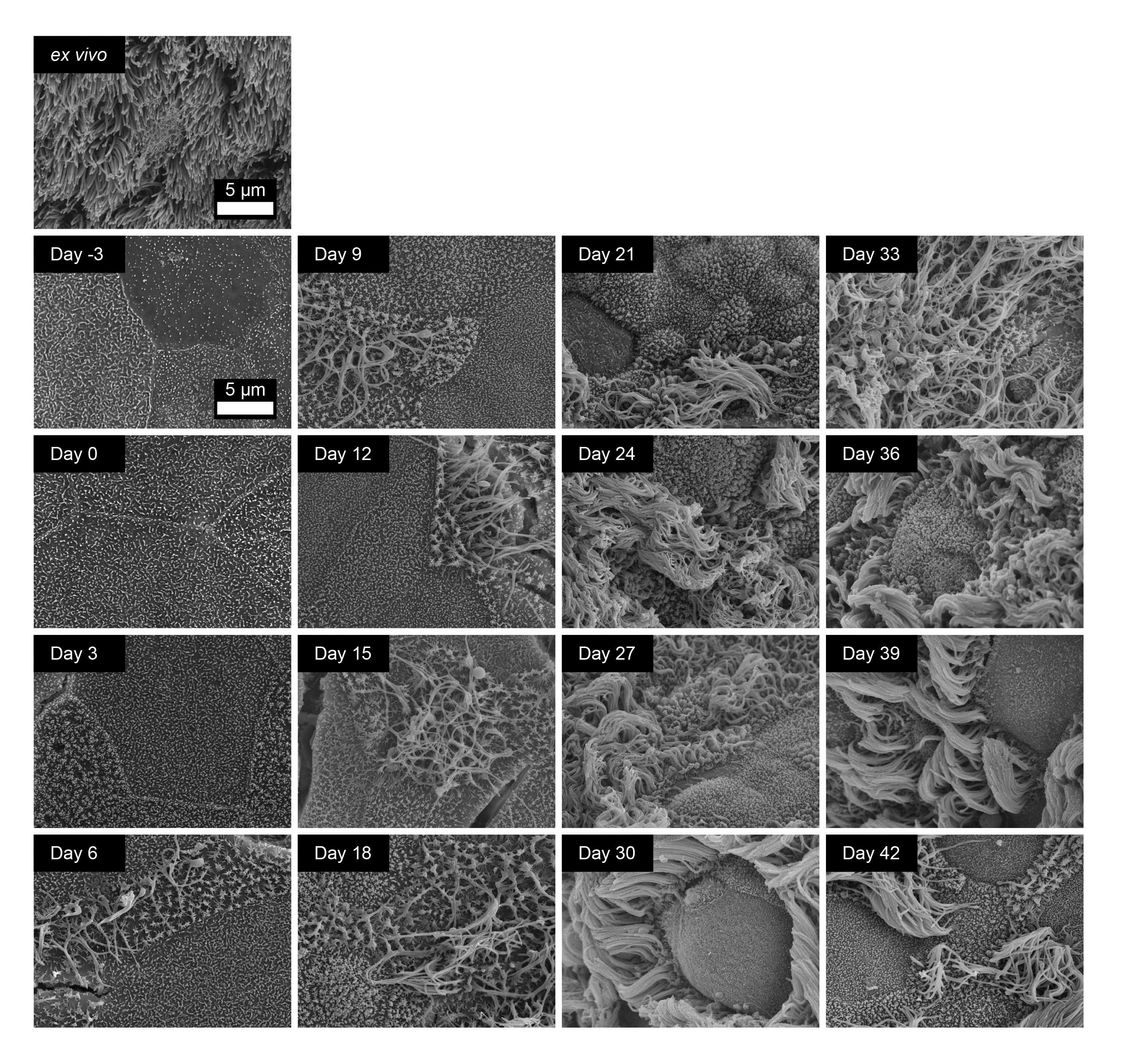

Supplement: S7 Fig — Ovine tracheal epithelial cell cultures were grown on cell culture inserts at an ALI and tissue layers at the indicated time points were fixed, processed and analysed by SEM. Ex vivo tissues were dissected prior to cell extraction and were also fixed, processed and analysed by SEM. (TIF) [file pone.0181583.s007.tif]

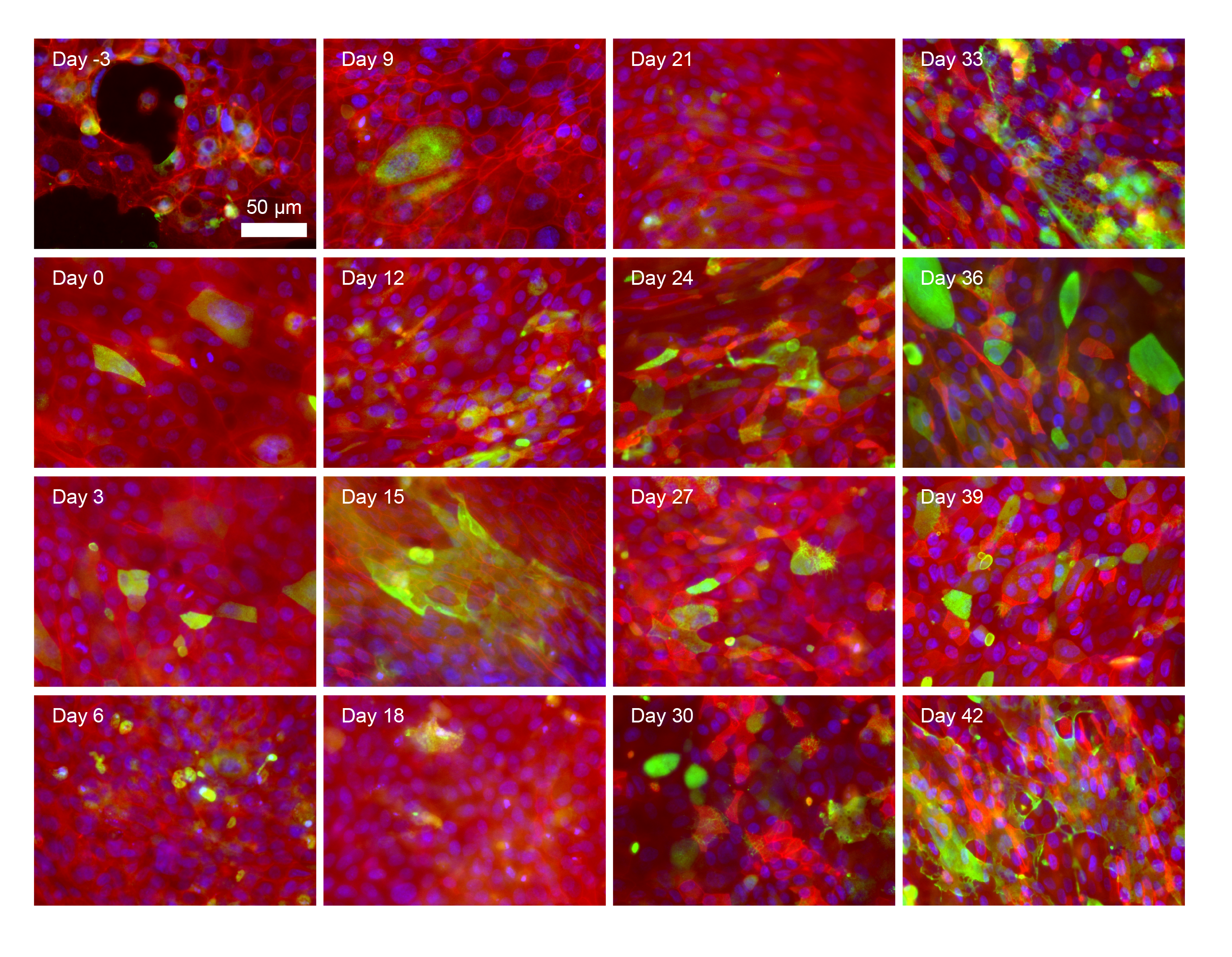

Supplement: S8 Fig — Ovine tracheal epithelial cell cultures were grown at an ALI for the indicated number of days (relative to establishment of the ALI), fixed and stained using jacalin-FITC to detect mucins (green) and rhodamine-phalloidin to stain the actin cytoskeleton (red). DAPI was used to stain nuclear DNA (blue). (TIF) [file pone.0181583.s008.tif]

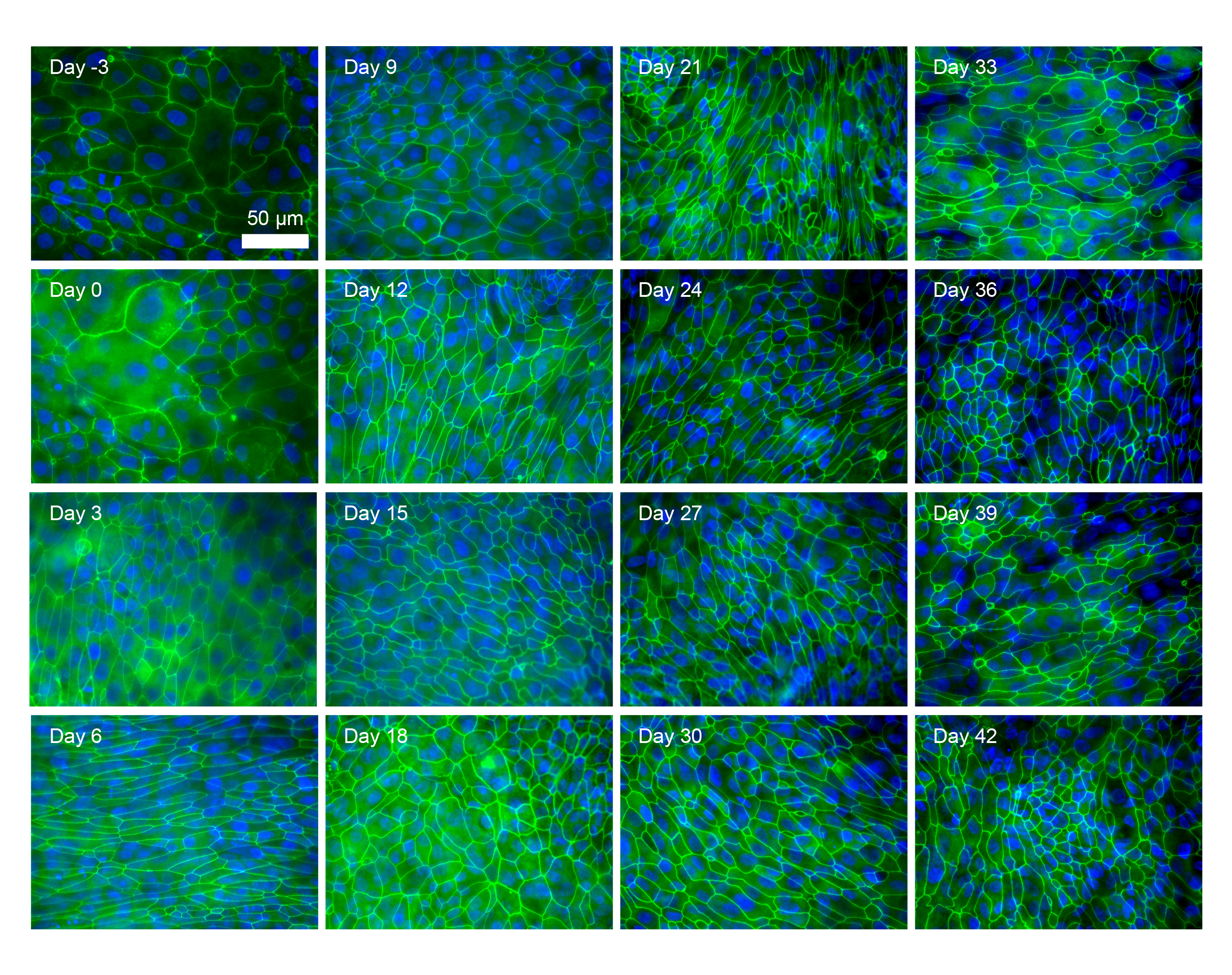

Supplement: S9 Fig — Ovine tracheal epithelial cell cultures were grown at an ALI for the indicated number of days (relative to establishment of the ALI), fixed and immunostained using an anti-ZO1 antibody (green). DAPI was used to stain nuclear DNA (blue). (TIF) [file pone.0181583.s009.tif]
